# Effect of guidewire insertion in fractional flow reserve procedure for real geometry using computational fluid dynamics

**DOI:** 10.1186/s12938-021-00935-y

**Published:** 2021-09-28

**Authors:** Yasser Abuouf, Muhamed AlBadawi, Shinichi Ookawara, Mahmoud Ahmed

**Affiliations:** 1 Department of Energy Resources Engineering, Egypt-Japan University of Science and Technology (E-JUST), Postal Code 21934, New Borg El-Arab City, P.O. Box 179, Alexandria, Egypt; 2 Mechanical Engineering Department, Faculty of Engineering, Alexandria University, Alexandria, Egypt; 3 Engineering Mathematics and Physics Department, Faculty of Engineering, Alexandria University, Alexandria, Egypt; 4 Department of Chemical Engineering, Graduate School of Science and Engineering, Tokyo Institute of Technology, O-okayama, Meguro-ku, Tokyo, 152-8552 Japan; 5 Mechanical Engineering Department, Assiut University, Assiut, 71516 Egypt

**Keywords:** Stenosis, Fractional flow reserve (FFR), CFD, Guidewire, Blood flow simulation, Non-Newtonian

## Abstract

**Background:**

Coronary artery disease is an abnormal contraction of the heart supply blood vessel. It limits the oxygenated blood flow to the heart. Thus, diagnosing its severity helps physicians to select the appropriate treatment plan. Fractional flow reserve (FFR) is the most accurate method to pinpoint the stenosis severity. However, inserting the guidewire across stenosis may cause a false overestimation of severity.

**Methods:**

To estimate the errors due to guidewire insertion, reconstructed three-dimensional coronary artery geometry from a patient-specific scan is used. A comprehensive three-dimensional blood flow model is developed. Blood is considered non-Newtonian and the flow is pulsatile. The model is numerically simulated using realistic boundary conditions.

**Results:**

The FFR value is calculated and compared with the actual flow ratio. Additionally, the ratio between pressure drop and distal dynamic pressure (CDP) is studied. The obtained results for each case are compared and analyzed with the case without a guidewire. It was found that placing the guidewire leads to overestimating the severity of moderate stenosis. It reduces the FFR value from 0.43 to 0.33 with a 23.26% error compared to 0.44 actual flow ratio and the CDP increases from 5.31 to 7.2 with a 35.6% error. FFR value in mild stenosis does not have a significant change due to placing the guidewire. The FFR value decreases from 0.83 to 0.82 compared to the 0.83 actual flow ratio.

**Conclusion:**

Consequently, physicians should consider these errors while deciding the treatment plan.

**Supplementary Information:**

The online version contains supplementary material available at 10.1186/s12938-021-00935-y.

## Background

The cardiovascular system permits blood to transport essential elements such as oxygen and nutrients through the body. The oxygenated blood leaves the left side of the heart through the aorta to the rest of the body. The coronary arteries start from the aorta and wrap around the heart. Small coronary branches go through the heart muscle to supply it with necessary oxygenated blood. Coronary artery disease (CAD) is the narrowing or blockage of the coronary arteries. Atherosclerosis is the main cause of CAD which is the buildup of plaques on the inner walls of the arteries. Plaques can reduce the blood flow to the muscles of the heart by physically clogging the artery [1]. The heart could starve oxygen and the vital nutrients which it needs to work properly without enough blood supply. This causes angina which is chest pain. A heart attack may occur if the blood supply to a portion of the heart muscle is cut off entirely [2]. High cholesterol levels, elevated blood pressure, diabetes mellitus (type 1 and 2), tobacco use, obesity, family history, and an unbalanced diet are identified as risk factors of CAD [3–5]. Treatment for CAD varies among lifestyle changes, drugs, or medical procedures [6]. The most appropriate treatment plan is determined by the physician according to the severity of the stenosis [2].

In recent years, several diagnostic methods have been developed to detect the location and severity of coronary stenosis. The most commonly used diagnostic techniques are qualitative in which diagnosing the stenosis severity depends on the physicians’ knowledge, expertise, and judgment. Thus, quantitative approaches are needed for more accurate severity prediction and consequently, better treatment plans can be provided. Intravascular ultrasound (IVUS) and fractional flow reserve (FFR) are the two most popular quantitative methods for stenosis diagnosis. The IVUS known as echocardiography is a medical imaging procedure that involves the use of a catheter with a miniaturized ultrasound transducer connected to the distal end. The catheter's other end is attached to a computerized ultrasound unit [7, 8]. As the IVUS can only provide 2-D images and cannot assess the functional significance of CAD [9], it is seldom used as a strict diagnostic tool. It uses the hydraulic diameters of the artery and the stenosis throat to predict the severity of the stenosis.

In the 1990s, the FFR approach was developed to assess the functional significance of stenoses in the epicardial coronary artery [10]. The ratio of the maximum myocardial blood flow in the presence of stenosis to the theoretical normal maximal flow in the same distribution is the value of FFR. In the case of minimal and continuous resistance, blood flow is proportional to pressure. As a result, blood pressure can be used as a proxy for blood flow. FFR is simply measured by dividing the stenosis's distal coronary pressure by the aortic pressure [11]. The FFR technique is invasive. As a result, scientists were working on new non-invasive diagnostic methods. Based on images obtained from coronary computed tomography angiography (CT) and simulation of blood flow using computational fluid dynamics (CFD), computed tomography fractional flow reserve (FFRCT) techniques are used to diagnose the severity of stenotic lesions. CT also shows the structure of the vessel as well as the presence or absence of atherosclerotic disease. FFRCT was found to be feasible and effective in diagnosing mild and severe stenosis [12]. Using various sets of boundary conditions and combining the effects of all simulations, Xie et al. [13] introduced a new CFD-based approach to diagnosing the severity of coronary stenosis. Kato et al. [14] devised a novel method for fluid analysis based on a one-dimensional mechanical model and CT image monitoring. The deformation variation of vessels and the volume variation of vessels were investigated to establish better boundary conditions [14]. Schrauwen et al. [15] used imaging data to present a new approach for estimating the pressure drop in human coronary arteries. The method works by gradually increasing the complexity of the geometry in three stages. FFRCT's cut-off value is 0.76, according to Dai et al. [16], and FFRCT is a reliable non-invasive tool for providing reliable functional information for coronary artery stenosis. However, there are still issues with the FFRCT mechanism that need to be addressed. Due to imaging resolution limitations, existing patient-specific imaging processing has difficulty reconstructing reliable 3-D models for vessels with a diameter smaller than 0.5 mm. Second, by using a multiscale simulation, it is important to determine patient-specific physiological parameters embodied in reduced-order models [17]. To predict or provide the mechanical properties of the coronary wall, sophisticated models and tests could be developed [9].

Invasive FFR, on the other hand, is now considered the most accurate technique for stenosis diagnosis. It is a guidewire-based technique that measures the stenosis's proximal and distal pressure (upstream and downstream) using a small sensor on the wire's tip [18, 19]. It is primarily used to determine the most effective treatment plan for moderate stenosis. Before angioplasty, the FFR clinical procedure is performed surgically by inserting a guide catheter (2.0–2.3 mm diameter) into the coronary ostia. Via the guide catheter, a sensor-tipped guidewire can be advanced. The guidewire is positioned along the stenosis to determine how much pressure is lost through the lesion [20]. High accuracy, instantaneous assessment of the severity of the stenosis, and simultaneous treatment with ballooning or stenting are all advantages of FFR. Unfortunately, the FFR's biggest drawback is that it is an invasive operation that necessitates an anesthetic incision. Additionally, inserting the guidewire across the stenotic lesion can result in a ‘tight-fit' between the affected vessel's lumen and the guidewire. This can cause an increase in the measured pressure drop, resulting in erroneous flow parameter estimation. As a result, FFR is ineffective as a diagnostic procedure [21–24].

Researchers modeled the fluid flow around the guidewire and numerically simulated the models to predict the flow field to improve the FFR procedure. As a result, the guidewire's optimum size and location were expected. Researchers have looked at different guidewire sizes. The flow parameters were found to be least affected by a guidewire with a diameter of 0.35 mm [25]. The effect of main arterial diameter on stenosis diagnosis procedure was investigated experimentally by Goswami et al. [26]. The effect of putting the guidewire on the magnitude of stenosis in idealized geometries was investigated in several studies [21, 22, 24]. Banerjee et al. [24, 27] investigated the effect of positioning the guidewire in various idealized stenosis and discovered that doing so causes the stenosis to be overestimated. The effect of adjusting the location and orientation of the guidewire on the measured pressure was investigated by Abuouf et al. [21–23], who discovered that the best position for the guidewire is at the centerline. Furthermore, Abuouf et al. [23] reported that when using a guidewire to diagnose stenosis severity, an error of 32% to 75% should be considered.

Based on the literature survey, all conducted researches in the assessment of invasive FFR using CFD are based on idealized simple geometry with the guidewire tip being after the stenotic region. In simple geometries, the flow is assumed to be a one-dimensional flow. Therefore, Simulating the blood flow in simplified geometries does not represent the real blood flow characteristics and will eventually lead to less accurate results. Moreover, to the authors’ knowledge, on the diagnosis of stenosis severity, there has been no reported research investigating the effect of placing guidewire using a real geometry. This research started by computationally simulating the flow during the FFR procedure for mild stenosis in real geometry using velocity inlet boundary condition [28]. It was found that simulating the model by placing the guidewire at a full position prior to the simulation leads to an overestimation of 34.3%. However, simulating the procedure of guidewire insertion is more accurate. It shows an overestimation of 7% [28]. Furthermore, the present study aims to investigate the effect of guidewire placement on the pressure measurements of FFR using a real geometry for different degrees of severity. In addition, a three-dimensional model is developed rather than the previously used one-dimensional model. Consequently, reconstructed patient-specific geometry with realistic pulsatile pressure inlet is modeled based on three-dimensional modeling and numerically simulated to investigate the mass flow rate and calculate FFR. Consequently, comparison with actual flow ratio is performed to estimate the error in diagnostic values due to guidewire placement.

## Results

To assess the effect of guidewire placement in FFR, the flow is simulated in patient-specific stenotic right coronary artery (RCA) without guidewire as a reference. The guidewire is simulated in each position for mild (40%, − *d* = 1.8 mm), moderate (65%, − *d* = 1.2 mm), and severe (85%, − *d* = 0.45 mm) stenoses. The effect of placing guidewire on stenosis diagnosis is studied by comparing the predicted FFR values with the actual flow ratio. In clinical procedure, the time-averaged pressure is only measured. In fact, it is clinically difficult to obtain the pressure values at a certain time. Based on the time-averaged pressure, FFR and pressure drop coefficient (CDP) can be obtained. So, the value of interest is the time-averaged pressure. Additionally, in the present study, the pressure profile is used at the inlet for accurate simulation. The calculated time-averaged pressure data were analyzed to determine the overall mean trans-stenotic pressure drop, CDP, and FFR values.

### Zero % stenosis (healthy artery)

Blood flow through a healthy artery is simulated for two reasons. The first reason is to predict the value of the flow rate of healthy artery as it is considered the reference value for FFR. The second reason is to calculate the constant C in Eq. 11 which is an important value to calculate FFR from pressure. This constant is calculated by dividing the time-averaged inlet pressure by the time-averaged pressure at the point after the stenosis in the stenosed artery. The C value is 5.14 and it will be used in calculating FFR in the stenosed artery from Eq. 11.

### ***Throat hydraulic diameter ***${{\varvec{d}}}_{{\varvec{m}}}=1.8\boldsymbol{ }{\varvec{m}}{\varvec{m}}$*** (40% stenosis)***

#### Variation of time-averaged pressure drop

**Fig. 1:**
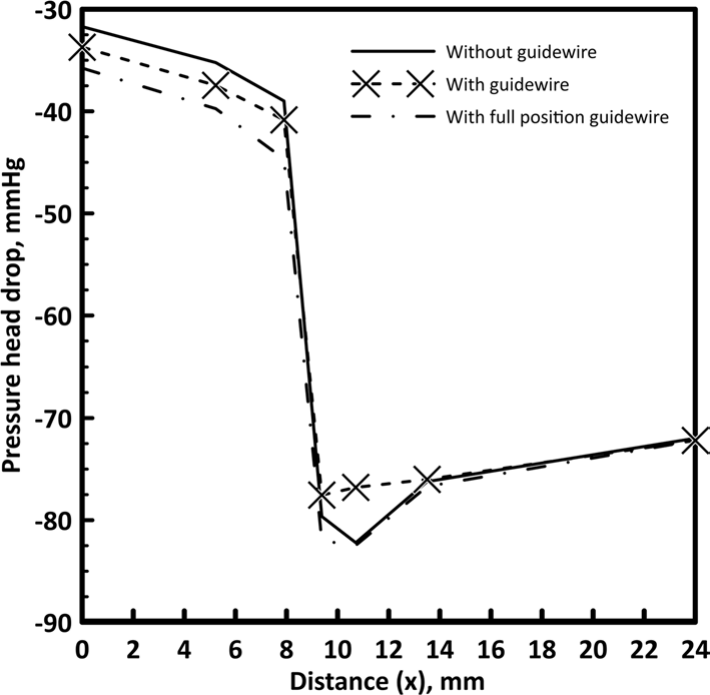
Pressure drop with and without the guidewire for 40% stenosis

Time-averaged pressure head drop values was analyzed for all guidewire state models (without guidewire, with full position guidewire, and with guidewire during insertion). The time-averaged pressure head drop with axial distance (*x*) is shown in Fig. 1. Placing the guidewire has a slight effect on the time-averaged pressure head drop. At the throat of the stenosis, the pressure reaches its minimum value then gradually increases again with increasing the area till reaching its original diameter. The pressure head with no guidewire decreases from 85.89 mmHg at the inlet by 31.7 mmHg to the beginning of the stenosis. With further reduction, the pressure head drop reaches 82.2 mmHg at the stenosis throat and then recovers by 10.22 mmHg before the bifurcation to reach a total pressure head drop of 71.98 mmHg. Placing the guidewire increases the pressure head drop at the beginning of the stenosis to 35.77 mmHg and slightly increases the pressure head drop at the stenosis throat to 82.4 mmHg at the minimum hydraulic diameter, and then the recovered pressure head becomes 10.27 mmHg to reach a total pressure head drop before the bifurcation of 72.19. Hence, the pressure head drop from the aortic pressure to the stenosis throat slightly increases from 82.2 mmHg to 82.4 mmHg due to the guidewire insertion.

However, considering the values of the pressure head drop when placing the guidewire in full position for all points, the pressure head drop increases to 33.72 mmHg at the beginning of the stenosis. Then, the pressure head drop reduces from the inlet to the throat by 77.6 mmHg then the pressure increases by 4.6 mmHg to a total pressure head drop of 72.19 as shown in Fig. 1. A comparison between the pressure head drop upstream, throat and downstream the stenosis is presented in Table 1. For the full position guidewire, the pressure head drop decreases because the flow rate is reduced due to the increase in resistance after the stenosis compared with the during insertion case.

**Table 1:** Pressure head drop at different points for 40% stenosis

	Upstream the stenosis	Stenosis throat	Downstream the stenosis
Without guidewire	− 31.7	− 82.2	− 72
Full position guidewire	− 35.8	− 82.5	− 72.2
During insertion guidewire	− 33.7	− 76.8	− 72.2

**Fig. 2:**
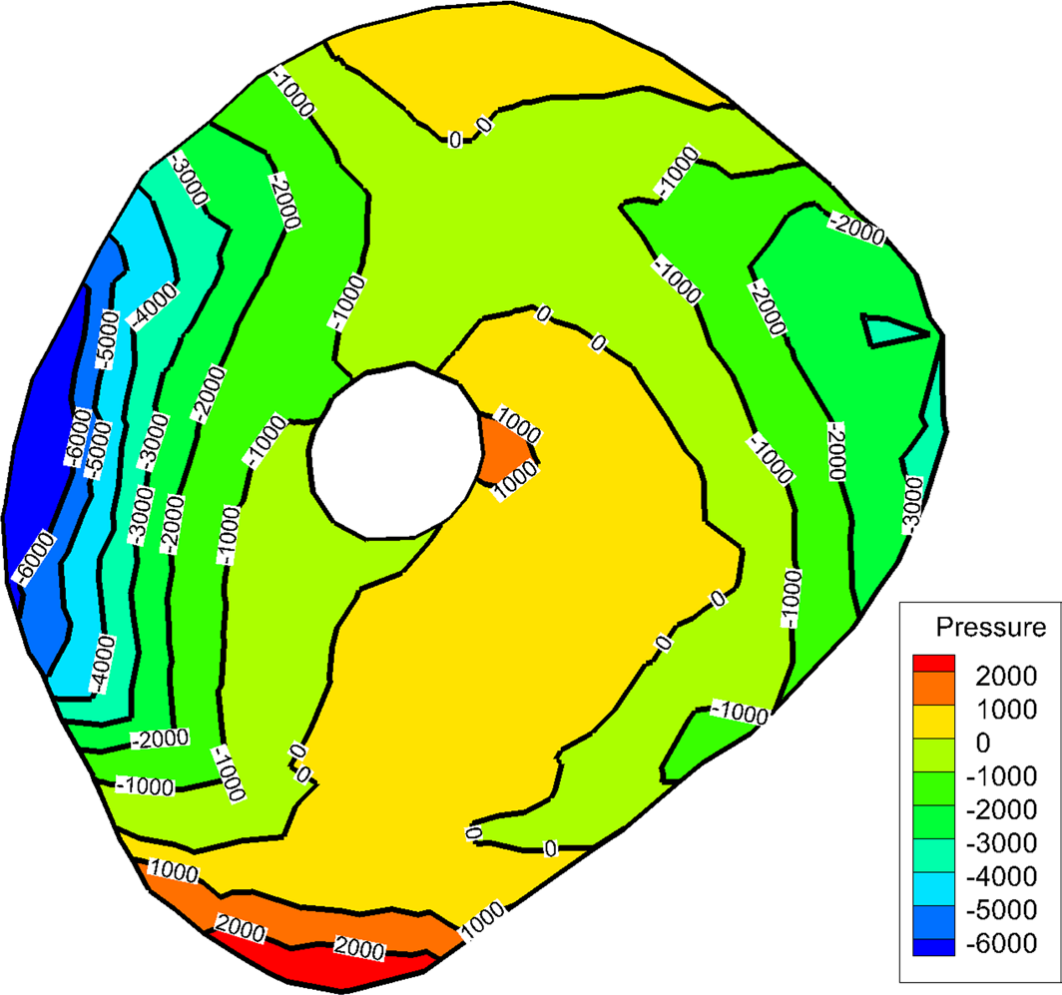
Pressure distribution at the throat cross-section of 40% stenosis (pressure in Pa)

Pressure values also depend on the position of the guidewire. The pressure over any cross-section is not constant as shown in Fig. 2 due to the consideration of all velocity components. Accordingly, the guidewire tip position at any cross-section has a significant effect on the value of the measured pressure.

### Flow rate and streamlines

**Fig. 3:**
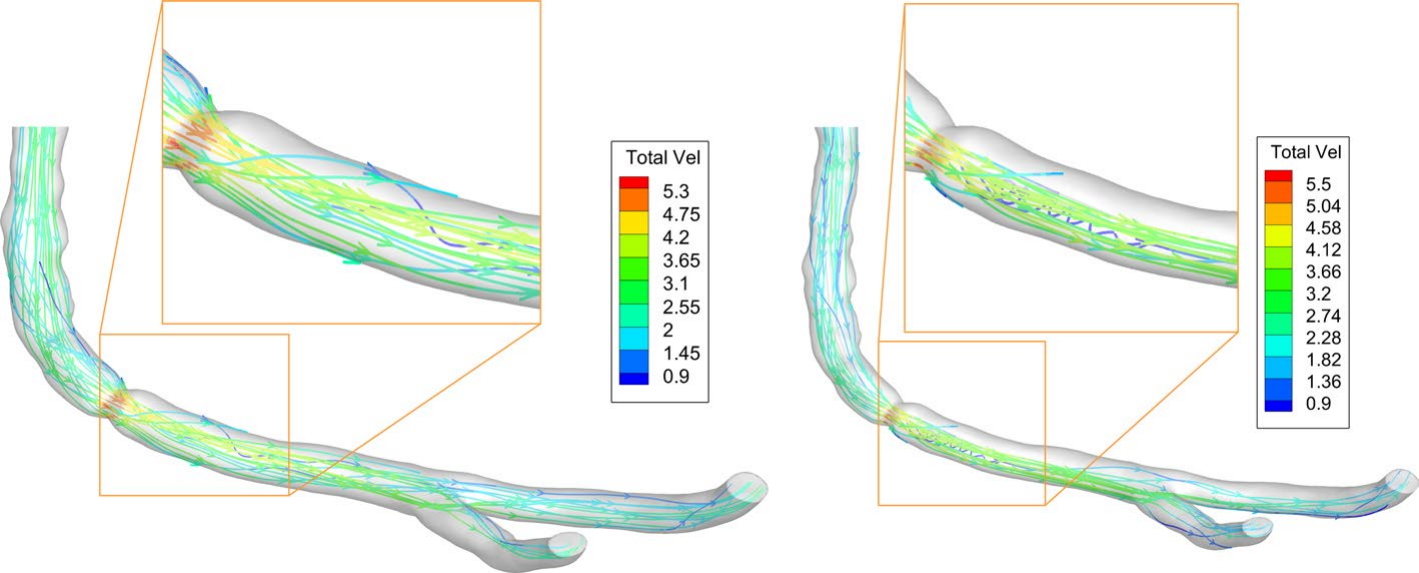
Streamlines at 40% stenosis. Left: without guidewire. Right: with guidewire (velocities in m/s)

The stenosis existence increases the total resistance in the RCA. Accordingly, the flow rate is reduced by 16.79% than that of the healthy artery which is calculated as follows: $\% reduction=\frac{{Q}_{\mathrm{Healthy}}-{Q}_{\mathrm{stenosis}}}{{Q}_{\mathrm{Healthy}}}\times 100$. The flow rate ratio between stenosed artery and healthy artery is 0.83. Inserting the guidewire reduces the flow rate entering the RCA. Due to the presence of the guidewire, the flow rate has further reduction by 5.92%. This flow is divided after the stenosis into two branches. The ratio of the flow entering the main branch changes. Before inserting the guidewire, 59% of the flow was directed to the main branch, however after inserting the guidewire, the velocity at the throat slightly increases guiding the flow through the main branch. Accordingly, the percentage slightly changes to 60.28%. Introducing the guidewire increases the formation of eddies after the stenosis as shown in Fig. 3. Hence, a slight increase in pressure head drop appears at the throat as presented in Fig. 1.

#### Pressure drop coefficient (CDP) and FFR

It is found that inserting the guidewire had a significant effect on the CDP as presented in Table 2. The value of CDP increases from 1.38. to 1.56 with an error of 13% due to the insertion of the guidewire. However, assuming that the guidewire is in full position before staring the simulation increases the CDP to 1.62 with an error 16.5% as shown in Table 2. This increase in the error is due to the change in the inlet velocity after placing the guidewire. Accordingly, it leads to an overestimation of the stenosis severity which matches the conclusions presented previously by Banerjee et al. [24] and Abuouf et al. [21, 22]. However, using CDP clinically is not applicable due to the difficulty in measuring the flow rate or velocity inside the coronary artery during the FFR procedure.

**Table 2:** Calculated variables for 40% stenosis

	$${P}_{a}$$$$(mmHg)$$	$${P}_{d}$$$$(mmHg)$$	$$\Delta p$$$$(mmHg)$$	Inlet average velocity (m/s)	CDP	Error in CDP	FFR	Error in FFR
Without	85.9	13.92	71.97	1.15	1.38	–-	0.83	–
During insertion	85.9	13.71	72.19	1.08	1.56	13%	0.82	1.2%
Full position	85.9	13.71	72.19	1.06	1.62	17.4%	0.82	1.2%

On the other hand, the FFR value depends only on the pressure measurements and represents the ratio between the flow in stenosed artery and the healthy artery. The flow ratio is calculated in this case and its value is found to be 0.83. During the guidewire insertion, The FFR value is 0.82. Accordingly, FFR is slightly more accurate and more applicable in stenosis diagnosis which contradict with Banerjee et al. [24] and Abuouf et al. [21–23] statements. They stated that CDP value is more accurate in stenosis diagnosis because it includes the inlet value in the calculations. The basis of such calculations was based on using constant inlet velocity which is not accurate. If the inlet velocity is known, the diagnosis process is complete and there is no need to measure pressure inside the artery. The main issue due to stenosis existence is the reduction in flow rate. Accordingly, the flow rate reduction is the target value in the diagnosis process and the pressure is measured to calculate this value.

### ***Throat hydraulic diameter ***${{\varvec{d}}}_{{\varvec{m}}}=1.2\boldsymbol{ }{\varvec{m}}{\varvec{m}}$*** (60% stenosis)***

#### Variation of time-averaged pressure drop

**Fig. 4:**
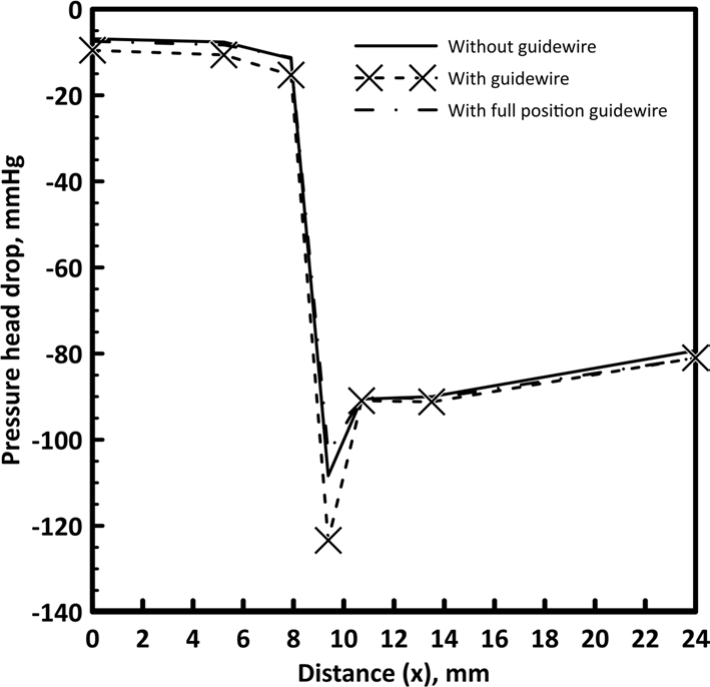
Pressure head drop with and without guidewire for 60% stenosis

Time-averaged axial pressure values were analyzed, and the overall time-averaged trans-stenotic pressure gradient is calculated for both guidewire states. The time-averaged pressure with axial distance (*x*) is shown in Fig. 4. Placing the guidewire has a remarkable effect on the time-averaged pressure head. At the throat of the stenosis, the pressure reaches its minimum value then gradually increases again with increasing the area till reaching its original diameter. The pressure head with no guidewire decreases from 85.89 mmHg at the inlet by 6.86 mmHg to the beginning of the stenosis. With further reduction, the pressure head drop reaches 108.39 mmHg at the stenosis throat and then increases by 11.25 mmHg before the bifurcation to reach a total pressure head drop of 79.31 mmHg. Placing the guidewire increases the pressure head drop at the beginning of the stenosis to 9.55 mmHg and slightly increases the pressure head drop at the stenosis throat to 123.8 mmHg at the minimum hydraulic diameter, and then the recovered pressure head becomes 9.95 mmHg to reach a total pressure head drop before the bifurcation of 81 mmHg. Hence, the pressure head drop from the aortic pressure to the stenosis throat slightly increases from 108.39 mmHg to 123.8 mmHg due to the guidewire insertion.

**Fig. 5:**
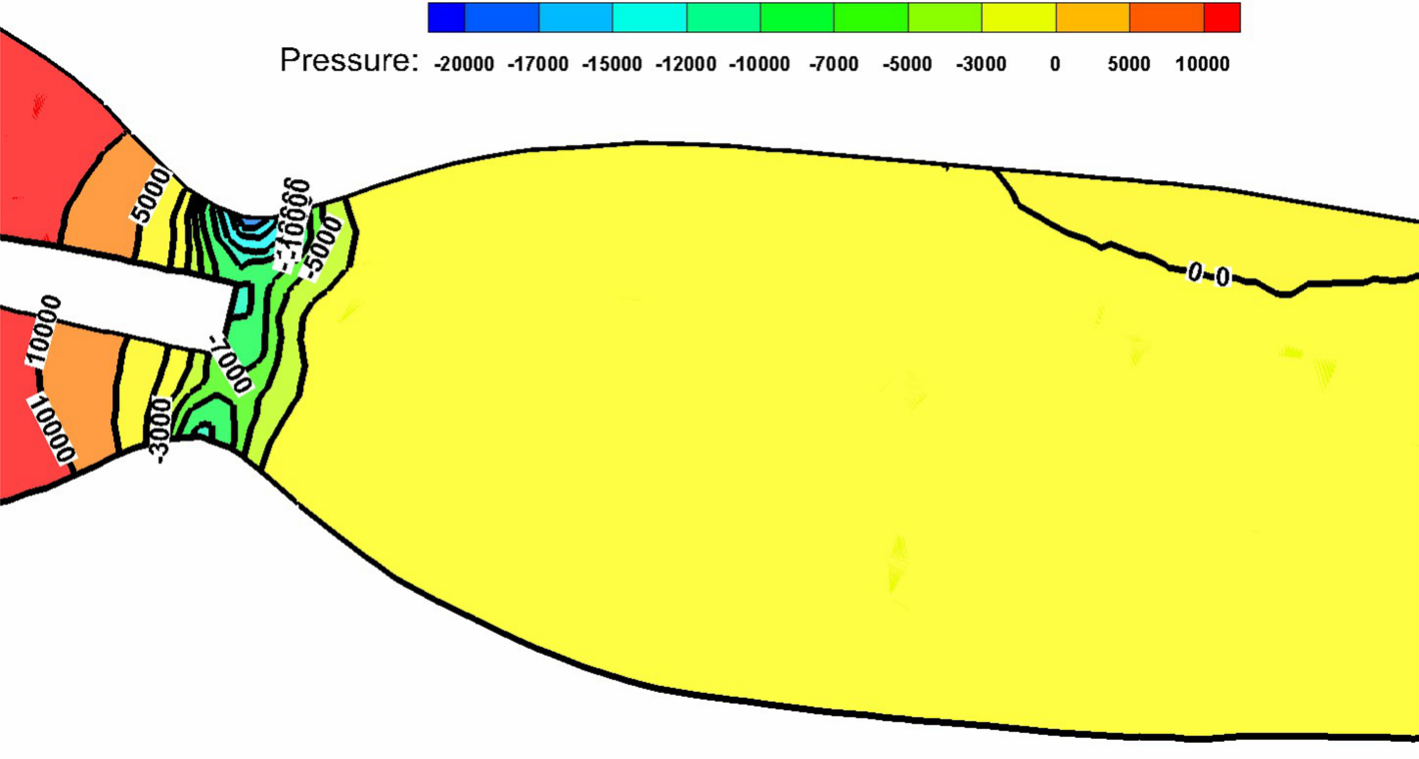
Pressure contour through 60% stenosis with guidewire (pressure in Pa)

The difference in pressure head drop values between the two cases which is shown in Fig. 4 is caused by shape of the stenosis. The geometry of the stenosis is sharp in convergence and divergence. Accordingly, the pressure changes rapidly from maximum (proximal side) to minimum at the throat then changes again to higher value distal the stenosis as shown in Fig. 5. It shows that the pressure changes between − 1.8 × 10^4^ Pa and 5 × 10^3^ Pa in less than 1 mm length.

However, considering the values of the pressure drop when placing the guidewire in full position for all points, the pressure head increases to 7.49 mmHg at the beginning of the stenosis compared to 6.86 mmHg for the without guidewire case. Then, the pressure head drop reduces from the inlet to the throat by 103.19 mmHg then the pressure increases by 9.23 mmHg to a total pressure head drop of 81 mmHg as shown in Fig. 4. A comparison between the pressure head drop upstream, throat and downstream the stenosis is presented in Table 3. The cross-sectional area of the flow decreases due to guidewire insertion which increases the resistance to the flow. Accordingly, the flow rate is reduced. Consequently, the reduction in flow rate compensates the increase in artery resistance. Therefore, the pressure head drop is almost the same for all cases.

**Table 3:** Pressure head drop at different points for 60% stenosis

	Upstream the stenosis	Stenosis throat	Downstream the stenosis
Without guidewire	− 6.9	− 108.4	− 79.3
Full position guidewire	− 9.6	− 123.4	− 81
During insertion guidewire	− 7.5	− 103.2	− 81

#### Actual flow rate ratio

The stenosis existence increases the total resistance in the RCA. Meanwhile, the pressure at the inlet and outlet are the same. Accordingly, the flow rate is reduced by 55.45% than the healthy artery. The flow rate ratio between stenosed artery and healthy artery is found to be 0.44. However, the flow rate ratio could be larger due to the flow supply from side branches which are neglected in this study. Gosling et al. [29] studied the effect of neglecting the side branches and found that the side branches support the main arteries with blood to compensate the reduction of the flow due to stenosis existence. Inserting the guidewire reduces the flow rate entering the RCA. Due to the presence of the guidewire, the flow rate has a further reduction by 7.93%. This flow is divided after the stenosis to the two branches. The ratio of the flow entering the main branch changes. Before inserting the guidewire, 69.16% of the flow was directed to the main branch. After inserting the guidewire, this percentage is slightly reduced to 67.77%. It is shown that the change in flow distribution between branches is not significantly affected by inserting the guidewire. However, if the distance between the stenosis and the bifurcation is reduced, the effect on flow distribution will increase.

#### Pressure drop coefficient (CDP) and FFR

As previously mentioned, FFR is more accurate and more applicable in stenosis diagnosis than CDP. The flow ratio is calculated in this case and its value is 0.44. The value of FFR is 0.33 when the guidewire is inserted while it is 0.43 in the case without guidewire as shown in Table 4. Accordingly, inserting the guidewire leads to overestimation of the stenosis which agrees with the previous study in simplified geometry by Abuouf et al. [21–23]. It was found that inserting the guidewire has a significant effect on the CDP as presented in Table 4. The value of CDP increases from 5.31 to 7.2 with an error of 35.6% due to the insertion of the guidewire. However, assuming that the guidewire is in full position before staring the simulation increases the CDP to 6.48 with an error 22%. The error in FFR value occurs because inserting the guidewire reduces the cross-sectional area which rises the resistance to the blood flow. Accordingly, the blood flow requires a higher pressure difference between upstream and downstream the stenosis which reduces the FFR value. Moreover, the error in CDP occurs due to the reduction in the inlet velocity. As well known, the CDP mainly depends on the value of the inlet velocity.

**Table 4:** Calculated variables for 60% stenosis

	$${P}_{a}$$$$(mmHg)$$	$${P}_{d}$$$$(mmHg)$$	$$\Delta p$$$$(mmHg)$$	Inlet average velocity (m/s)	CDP	Error in CDP	FFR	Error in FFR
Without	85.9	6.59	79.31	0.62	5.31	–	0.43	–
During insertion	85.9	4.91	80.99	0.56	7.2	35.6%	0.33	23.2%
Full position	85.9	4.91	80.99	0.57	6.48	22%	0.33	23.2%

### Throat hydraulic diameter ${{\varvec{d}}}_{{\varvec{m}}}=0.45 {\varvec{m}}{\varvec{m}}$ (85% stenosis)

#### Variation of time-averaged pressure drop

**Fig. 6:**
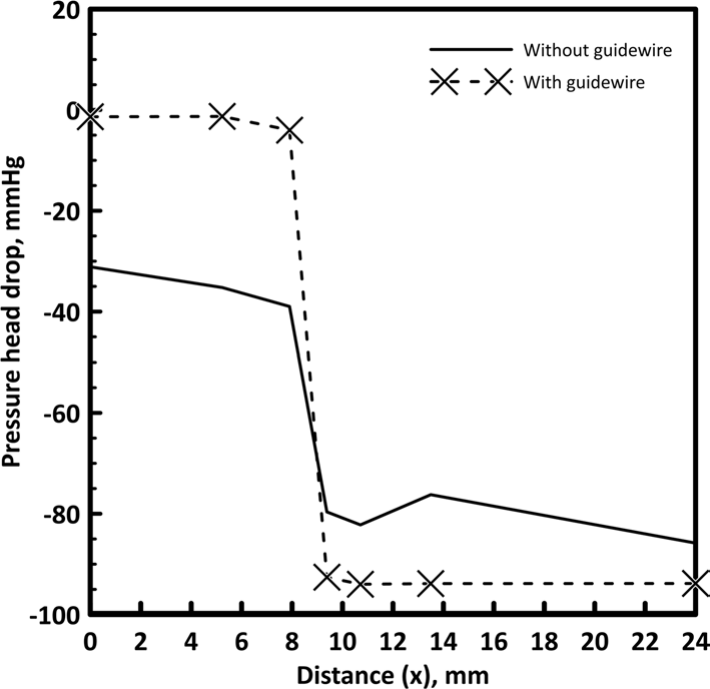
Pressure drop with and without guidewire for 85% stenosis

In the same context of 60% stenosis, the time-averaged pressure with axial distance (*x*) is shown in Fig. 6. In this case, placing the guidewire has a substantial effect on pressure head. The pressure head is normal in case without inserting the guidewire. The pressure head with no guidewire decreases from 85.89 mmHg at the inlet by 31.12 mmHg to the beginning of the stenosis. With further reduction, the pressure head drop reaches 82.2 mmHg at the stenosis throat and then increases by 9.68 mmHg before the bifurcation to reach a total pressure head drop of 85.81 mmHg. However, when the guidewire is placed the pressure head drop is reduced in both sides of the stenosis with large pressure head drop at the throat region. The pressure head drop is 1.36 mmHg to the beginning of the stenosis then reaches to 92.76 mmHg at the end of the throat. The reason for this effect is that the guidewire is in a tight-fit setting with the stenosis and causes blockage to the blood flow. Accordingly, the flow rate is significantly reduced as will be discussed in the next section.

#### Actual flow rate ratio

The stenosis existence increases the total resistance in the RCA. The cross-sectional area is reduced from 11.12 mm^2^ to 0.159 mm^2^. Accordingly, the flow rate is reduced by 92.18% than the healthy artery. The flow rate ratio between stenosed artery and healthy artery is 0.078. Inserting the guidewire causes a severe blockage in the artery. Accordingly, the flow rate has a further reduction by 43.37%. The reduction in flow rate when placing the guidewire compared with the healthy artery is 95.57%. Hence, FFR procedure is not recommended to be used in severe CAD cases and this was recommended by Rabbat et al. [30].

#### Pressure drop coefficient (CDP) and FFR

The flow ratio is calculated in this case and its value is 0.076. The value of FFR is 0.0012 when the guidewire is inserted while it is 0.078 in the case without guidewire as shown in Table 5. It was found that inserting the guidewire had a significant effect on the CDP as presented in Table 5. The value of CDP increases from 185 to 635 with an error of 243% due to the insertion of the guidewire. However, assuming that the guidewire is in full position before staring the simulation increases the CDP to 2632. The significant change in value between the cases is due to the severe blockage of the artery. The two cases of guidewire have different values because the velocity used in calculating the CDP is the average value for all positions in the case simulating the insertion. However, it is only one value in full position case, and it is low due to the blockage in the artery. In previous studies, the flow was forced through the artery by assuming inlet velocity boundary condition. In this study, the inlet pressure was not sufficient to allow the flow to pass through the stenosis with the guidewire.

**Table 5:** Calculated variables for 85% stenosis

	$${P}_{a}$$$$(mmHg)$$	$${P}_{d}$$$$(mmHg)$$	$$\Delta p$$$$(mmHg)$$	Inlet average velocity (m/s)	CDP	FFR
Without	85.9	0.75	85.15	0.108	185	0.076
During insertion	93.9	0.022	93.88	0.061	635	0.0012
Full position	93.9	0.022	93.88	0.03	2632	0.0012

## Discussion

The previous assessments for invasive FFR procedure using CFD were found to be inaccurate due to considering several assumptions such as simplifying the geometry and specifying the velocity at the inlet. Thus, in the current study, several assumptions are modified including geometry selection, blood model and boundary conditions, which in turn led to more realistic results.

The geometry in Banerjee et al.’s [24] work was a simplified 2-D axisymmetric, placing the guidewire in full position. The flow is assumed one-dimensional flow. Abuouf et al. [23] modified the geometry to a 3D, one-directional flow with full position guidewire. The guidewire was placed in different positions and different angles. For this study, the geometry was obtained from a real CT scan, with a better in-steps guidewire insertion process simulation.

In addition, Banerjee et al. [24] established an experimental setup for simulating the blood flow in the stenotic artery and used Carreau’s model in the computational simulation. Abuouf et al. [23] used the same experimental results to validate the 3D blood flow model. In this study, five blood models were validated against clinical measurements rather than experimental data, however, the latter validation proved that Carreau’s model is the most appropriate model to simulate the blood flow for this case.

Boundary conditions of the previous works of Banerjee et al. [24] and Abuouf et al. [23] were based the diagnosis on the CDP value which relies on the blood’s flow average velocity. This is not applicable, as the blood flow average velocity cannot be obtained clinically, and that is why the guidewire is used to diagnose stenosis severity, so using FFR is clinically more applicable, as it relies on the pressure values to estimate the flow ratio. Consequently, Banerjee et al. [24] and Abuouf et al. [23] used velocity inlet as a boundary condition. However, this study can only use the pressure inlet as a boundary condition which makes it more applicable with clinical data.

The error estimation of both Banerjee et al. [24] and Abuouf et al. [23] was based on comparing the CDP values using guidewire to that without guidewire. In this study, the error estimation was based on comparing the FFR values to the actual flow ratio. In Banerjee et al.’s [24] study, the maximum error values in mild, moderate and severe stenosis are 37%, 42%, and 73%, respectively. In Abuouf et al. [23] study, the mild and the moderate maximum error values were with values of 52% for mild and 76% for moderate. However, it was less in the severe stenosis case with a value of 58%. However, in this study, the values were less than both Banerjee et al. [24] and Abuouf et al. [23] studies for mild and moderate stenosis cases with values of 1.2% and 23%, respectively. In addition, no results for the severe stenosis case can be obtained because the artery was blocked by the guidewire which is realistic as the guidewire is not applicable for severe stenosis. The main reason for the noticeable reduction in maximum error is the criteria of calculating the error. In the present study, the error is compared with the ratio between the flow in stenotic artery to the flow in healthy artery. In the other hand, all previous studies compared the CDP values with guidewire to the values without guidewire.

As far the authors’ knowledge, the present work is the first investigation related to the effect of inserting the guidewire on FFR and CDP values. It is considered a real patient-specific geometry obtained from CT scan. In addition, realistic inlet pressure boundary conditions is applied compared with the previously used inlet velocity that neglected the effect the stenosis existence in flow rate reduction. Additionally, the current study compared FFR and CDP values between placing the guidewire in full position and simulating the insertion process of the guidewire by placing it at different locations through the stenosis. On the other side, several assumptions are considered in the current study. First, the arterial tree is not complete which leads to neglecting the resistance of part of the arteries. Thus, it is recommended to use patient-specific full geometries or get patient-specific outlet boundary conditions. Second, the arterial wall is assumed to be rigid. The elasticity of the wall should be taken into consideration using fluid structure interaction (FSI). Finally, the insertion length of the guide wire and the location and the shape of the stenosis are suspected to have a significant impact on the values of FFR and CDP. Thus, the ongoing research work of our group is currently searching for the effects of these factors on FFR and CDP.

## Conclusion

Fractional flow reserve (FFR) is the most accurate procedure for stenosis diagnosis. FFR has an instantaneous estimation of the severity of the stenosis. However, inserting the guidewire across the stenotic lesion causes a ‘tight-fit’ between the lumen of the affected vessel and the guidewire. This can lead to a false estimation of the flow parameters. The effect of inserting the guidewire was investigated on the accuracy of stenosis diagnosis in real geometry with realistic boundary conditions. In the moderate stenosis, it was found that placing the guidewire leads to an overestimation of severity. At an actual flow ratio of 0.44, the FFR is found to be 0.43 in case of no guidewire while it reduced to be 0.33 by using guidewire with an error of 23.26%. Besides, inserting the guidewire reduced flow rate by 7.93%. On the other hand, the CDP increases from 5.31 without guidewire to 7.2 due to guidewire insertion with an error of 35.6%. In the mild stenosis, there is no significant change in the FFR values due to placing the guidewire where it decreases from 0.83 to 0.82. In addition, the flow rate is reduced by 5.9% due to the insertion of guidewire. However, the CDP increases from 1.38 to 1.56 with an error of 13%. In case of 85% hydraulic diameter ratio which is considered as a severe stenosis, inserting the guidewire blocks the flow and reduces the flow rate by 95.57%. Accordingly, it is not recommended to use invasive FFR procedure in severe stenosis. The physician should consider these errors when using FFR in stenosis diagnosis specially in moderate stenosis.

## Methods

### Physical model

**Fig. 7:**
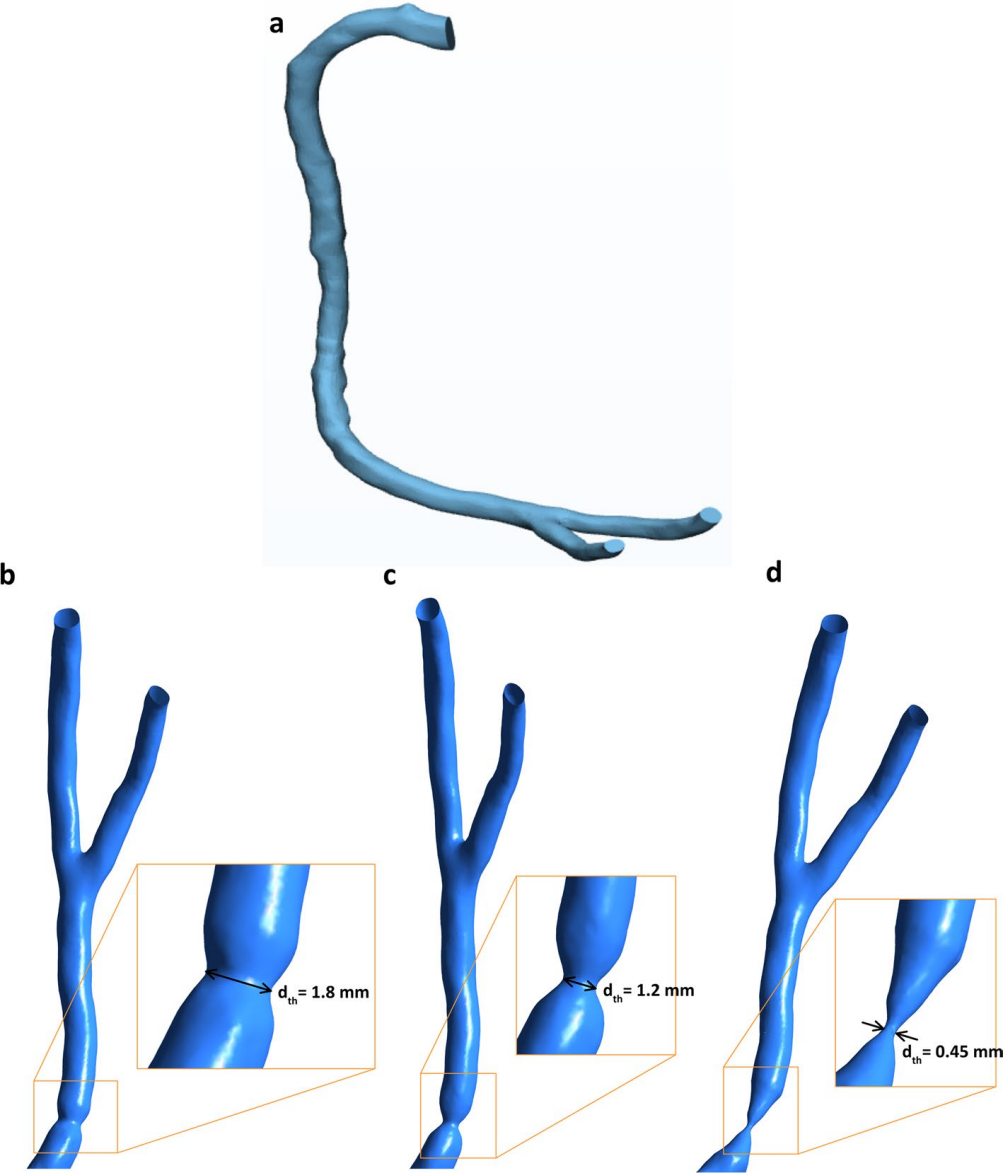
Geometry of RCA with three hydraulic diameter ratios. a Healthy artery. b d_th_ = 1.8 mm (40%). c d_th_ = 1.2 mm (60%). d d_th_ = 0.45 mm (85%)

In the present work, the procedure of invasive FFR to diagnose stenotic right coronary artery (RCA) is assessed using CFD. To get a real geometry and define the computational domain, anatomic data of healthy RCA were obtained from a clinically indicated CT angiogram. The CT develops images on Digital Imaging and Communication in Medicine (DICOM) format. Each one of the DICOM images of the CT approach represents a “slice” in the patient’s body. To obtain a 3-D reconstruction of the artery, the slices of all planes are stacked, then the spaces between them are interpolated, forming a volume [31]. The projection images are reconstructed into 3-D geometry. To reduce the abnormalities and imperfections resulted from the segmentation process, the 3-D geometry of the healthy RCA is imported to a trial version of 3-Matic software. Softening and refining the geometrical elements will allow working in a more efficient way as shown in Fig. 7a.

Moreover, three stenoses with different throat hydraulic diameters, which is calculated by $d=\frac{4\times \mathrm{Area}}{\mathrm{wetted perimeter}}$, and a 0.35-mm guidewire are developed in the geometry using 3-Matic software. The approximate centerline is specified using 3-matic software. Accordingly, the guidewire is placed at the defined centerline. The reduction percentages in hydraulic diameter $\frac{{d}_{\mathrm{artery}}-{d}_{\mathrm{throat}}}{{d}_{\mathrm{artery}}}\times 100$ are 40%, 60% and 85% representing mild, moderate, and severe stenoses, respectively, as shown in Fig. 7b–d. These values are selected based on the Society of Cardiovascular Computed Tomography (SCCT) guidelines [32].

**Fig. 8:**
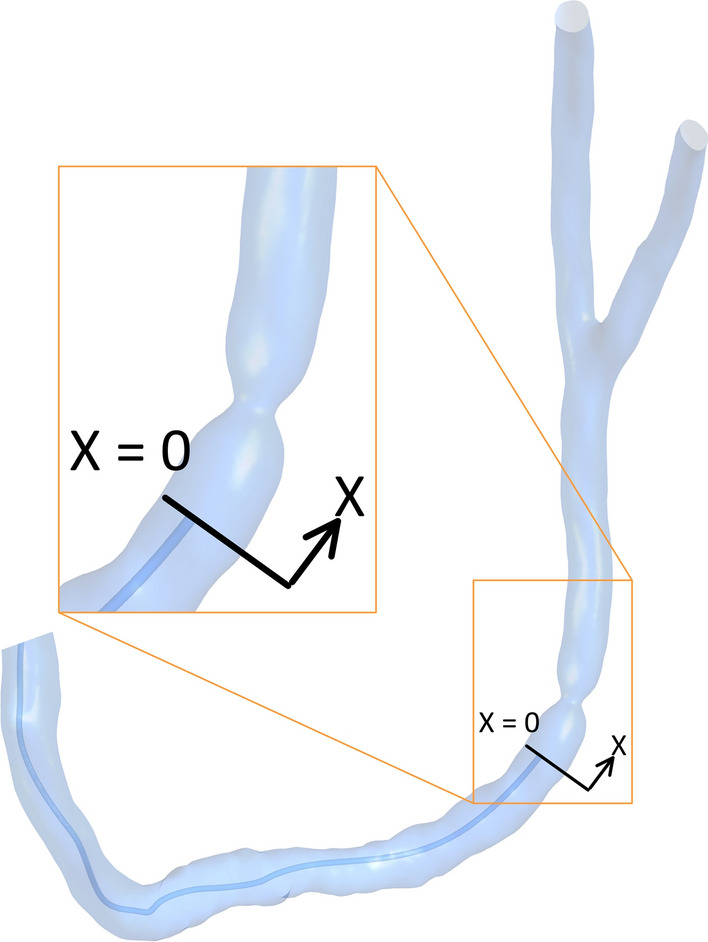
Guidewire first position placement in case of 60% stenosis

The guidewire is placed in seven positions to imitate the wire inserting process to simulate the real clinical procedure. The first position is placing the tip of the guidewire before the stenosis at a distance 60 mm from the inlet which is considered *x* = 0 as presented in Fig. 8. The other positions are placing the tip at different distances in the direction of the flow (*x*) through the stenosis and after the stenosis to predict the pressure through the lesion. These positions are at *x* = 5.3 mm, *x* = 8 mm, *x* = 9.4 mm, *x* = 10.7 mm, *x* = 13.5 mm, and *x* = 24 mm. These positions are distributed as follows: two are placed before the stenosis throat, two are at the throat and two positioned after the throat.

### Theoretical analysis

To evaluate the FFR procedure, blood flow into a stenotic coronary artery with and without a guidewire must be simulated. To establish the governing equations of blood flow in the stenosis with guidewire, the following assumptions are taken into consideration.

The flow is incompressible. Erythrocytes (red blood cells), which carry oxygen and carbon dioxide to and from the organs, leukocytes (white blood cells), which are part of the immune system, and thrombocytes (platelets), which are important for blood clotting, are among the cellular blood components. Plasma constitutes approximately 55% of the blood; the remaining 45% is erythrocytes. Leukocytes and thrombocytes represent a very small percentage of the blood. The density of the plasma is $1025 \mathrm{kg}/{\mathrm{m}}^{3}$; the existence of erythrocytes increases the density to $1050\mathrm{ kg}/{\mathrm{m}}^{3}$ [1].

- The flow is incompressible with density of 1050 kg/m^3^.
- As the value of Reynolds number does not exceed 2000, the flow is laminar. Several researchers stated that the flow might be turbulent at Reynolds number lower than 2000 [33, 34]. In addition, Cho et al. [35] presents a critical Reynolds number value of 500, and defines the critical Reynolds number as the value at which flow separation occurs in the divergent part of the stenosis. However, this does not mean that the flow has turned turbulent [36]. The existence of eddies does not require a turbulence model to simulate the laminar flow as presented in a review article about simulating blood flow in cerebral arteries [37].
- Plasma constitutes approximately 55% of the blood. As well known, plasma is a Newtonian fluid, but the existence of red blood cells changes the behavior of the blood to be a non-Newtonian flow [38].

#### Governing equations

The mass and momentum conservation equations for a non-Newtonian incompressible fluid can be written as

1 $$\nabla .\mathrm{V}=0,$$

2 $$\rho \frac{DV}{Dt}=\rho g+\nabla .{\tau }_{ij},$$

where V is the velocity vector; g is the vector acceleration of gravity; $\rho$ is the density, and ${\tau }_{ij}$ is the stress tensor. The body force is neglected (*g* = 0) [39]. The stress tensor can be represented as follows:

3 $${\tau }_{ij}=\left[\begin{array}{ccc}{\tau }_{xx}& {\tau }_{xy}& {\tau }_{xz}\\ {\tau }_{yx}& {\tau }_{yy}& {\tau }_{yz}\\ {\tau }_{zx}& {\tau }_{zy}& {\tau }_{zz}\end{array}\right].$$

The blood is a suspension of erythrocytes in plasma. Consequently, blood properties depend mainly on the volume fraction of erythrocytes in the plasma. The exact composition of the blood is the main factor to consider while selecting an appropriate non-Newtonian model. In the present study, five non-Newtonian models are used in simulating the blood flow and compared with the clinical measurements. The Carreau model is selected based on minimum differences between measured and predicted data. Detailed results and analysis of all tested models are included in Additional file 1: Appendix S1. Based on Carreau model, the relation between the dynamic viscosity ($\mu )$ and strain rate ($\dot{\gamma }$) can be stated as [38]:

4 $$\mu ={\mu }_{\infty }+\left({\mu }_{o}-{\mu }_{\infty }\right)\times {\left[1+{\left(\lambda \dot{\gamma }\right)}^{2}\right]}^{\frac{n-1}{2}}.$$

The coefficients for Carreau model could be written as follows: the value of zero shear rate viscosity, *μ*_0_ = 0.055 Pa.s, the infinite shear rate viscosity, *μ*_∞_ = 0.00339 Pa.s; time constant, *λ* = 9.56 s; and the power index, *n* = 0.2 [40].

The main parameters of interest are the pressure drop along the stenosis, FFR, and CDP which is defined as the ratio of the trans-stenotic drop of the mean pressure to the proximal dynamic pressure [28]:

5 $${\mathrm{CDP}}_{ }=\frac{\Delta {\tilde{p }}_{ }}{0.5\rho {\stackrel{\sim }{\overline{u}} }_{e}^{2}},$$

where *ρ* is the blood density and ${\stackrel{\sim }{\overline{u}} }_{e}^{2}$ is the spatial and temporal mean blood velocity in the proximal vessel.

The CDP is a dimensionless quantity that represents the fluid flow resistance caused by a pressure drop in a blood vessel. The CDP depends primarily on the stenosis geometry, flow rate, and velocity variation with time. The CDP value is not limited within a small range; an accurate cut-off value can be obtained for the CDP after human clinical trials. The CDP is considered a viable diagnostic parameter used in clinical settings [24, 41]. However, it is clinically hard to obtain the proximal velocity during the procedure.

**Fig. 9:**
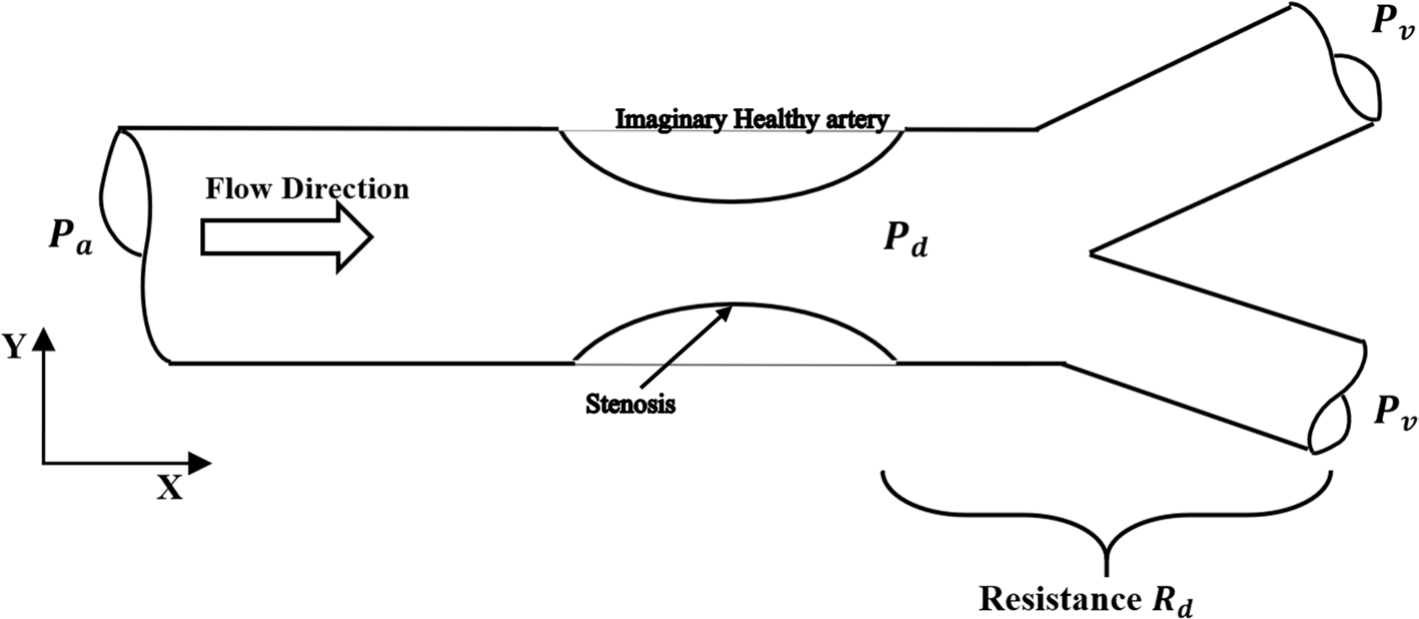
Artery schematic

The FFR value is defined as the ratio of the maximal myocardial blood flow in the presence of a stenosis to the theoretical maximal flow in the same distribution in healthy artery, and it is represented by

6 $$\mathrm{FFR}=\frac{{Q}_{S}}{{Q}_{H}},$$

where ${Q}_{H}$ is the healthy artery flow rate and ${Q}_{s}$ is the stenotic flow rate. To deduce the relation between the flow ratio (FFR) and the pressure measurements, a simplified structure represents blood flow in healthy and stenotic artery is shown in Fig. 9. The flow starts with aortic pressure ${P}_{a}$ and ends with venous pressure ${P}_{v}$. The healthy artery from the aortic to venous pressure has an equivalent resistance to the flow represented by ${R}_{H}$. In the stenotic artery, the resistance after the stenosis has equivalent value ${R}_{d}$. The velocity at the beginning and the end are neglected.

In the case of healthy artery, the pressure drop could be calculated using Darcy equation for losses in pipes assuming that there is no collateral flow occurring as,

7 $${P}_{a}-{P}_{v}=\frac{\rho fl{v}^{2}}{2d}=\frac{64\rho l{v}^{2}}{2Re d}=\frac{32\mu lv}{{d}^{2}}=\frac{128\mu l{Q}_{H}}{\pi {d}^{4}}={R}_{H}{Q}_{H},$$

where ${P}_{a}$ is the aortic pressure; ${P}_{v}$ is the central venous pressure and ${R}_{H}$ is the resistance of the healthy artery.

For the stenotic artery, the pressure drop distal to the stenosis is written as:

8 $${P}_{d}-{P}_{v}={R}_{d}{Q}_{s},$$

where ${P}_{d}$ is the distal pressure and ${R}_{d}$ is the resistance in the distal artery.

Dividing Eq. (8) by Eq. (7) results in:

9 $$\frac{{P}_{d}-{P}_{v}}{{P}_{a}-{P}_{v}}=\frac{{R}_{d}}{{R}_{H}}\frac{{Q}_{s}}{{Q}_{H}}.$$

Hence the central venous pressure is always zero gauge [42], the equation will be as follows:

10 $$\frac{{Q}_{s}}{{Q}_{H}}=\frac{{P}_{d}}{{P}_{a}}\frac{{R}_{H}}{{R}_{d}},$$

11 $$\frac{{Q}_{s}}{{Q}_{H}}=C\frac{{P}_{d}}{{P}_{a}}.$$

The pressure measurement during invasive FFR procedure is performed under hyperemia condition (maximum vasodilatation) to make sure that the resistances ${R}_{d}$ and ${R}_{H}$ are constant and minimal [10]. Hence, the value C is constant and it could be calculated from the simulations by applying Darcy equation between the imaginary distal point (${P}_{d}^{^{\prime}}$) and the end of the artery in healthy case:

12 $${P}_{d}^{\mathrm{^{\prime}}}-{P}_{v}={R}_{d}{Q}_{H}.$$

It should be noted that the value ${R}_{d}$ is the same in both cases (healthy and stenotic). Accordingly, the value C is represented as:

13 $$C=\frac{{P}_{a}}{{P}_{d}^{\mathrm{^{\prime}}}}.$$

Hence, the flow is pulsatile in actual conditions, the pressure values used in Eqs. 11 and 13 are the time-averaged pressure calculated by:

14 $${P}_{\mathrm{mean}}=\frac{{\int }_{0}^{T}Pdt}{T},$$

where $T$ is the time of the cardiac cycle, and $P$ is the required pressure at any point.

#### Boundary and initial conditions

**Fig. 10:**
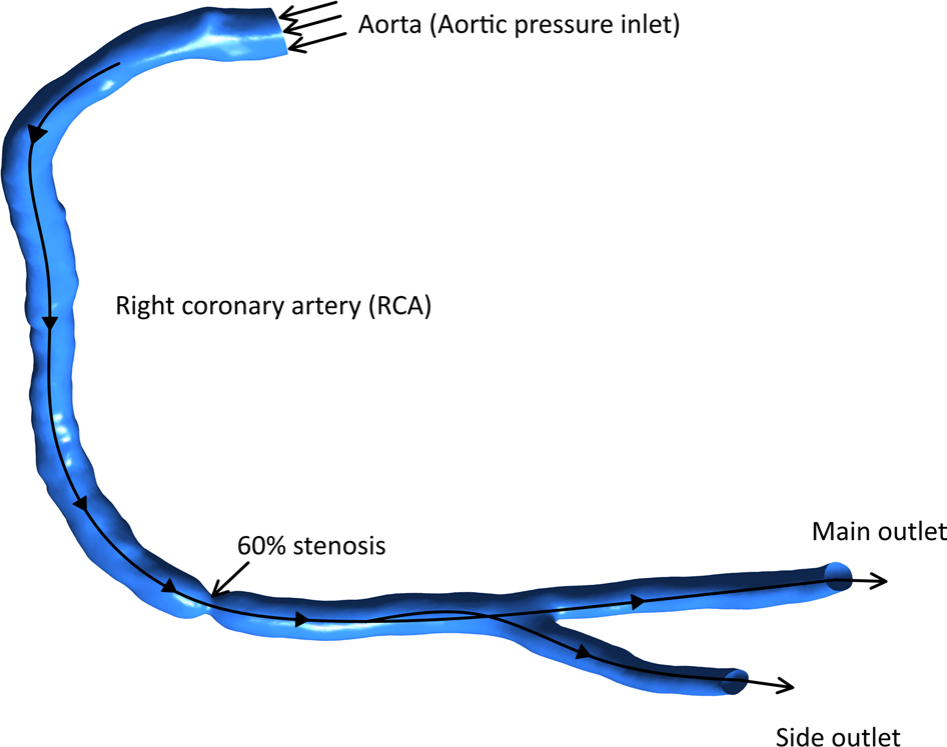
Stenotic RCA with 60% stenosis

To solve the governing equations, boundary conditions must be specified at the blood vessel wall, inlet and outlet of the artery as shown in Fig. 10.

**Fig. 11:**
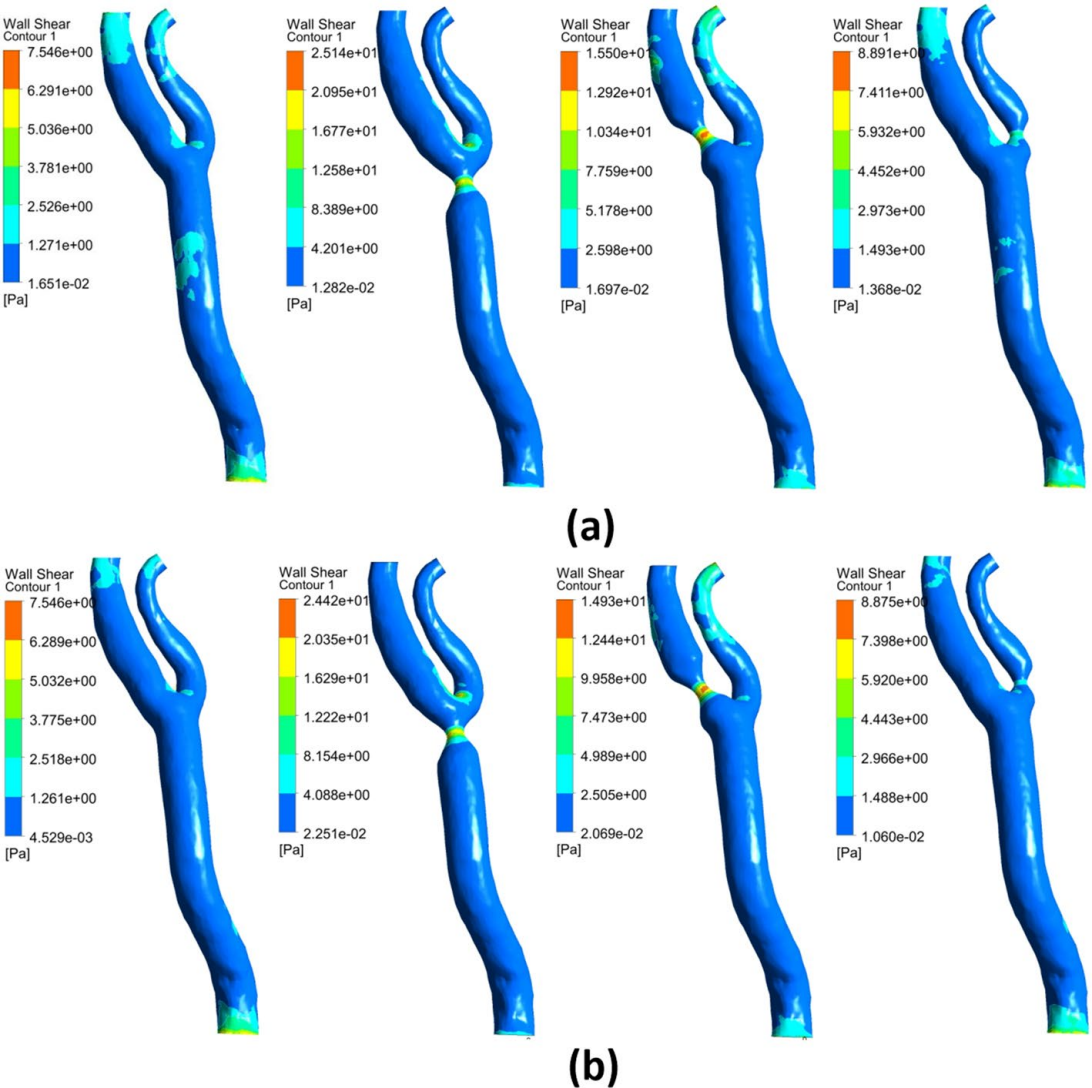
Wall shear stress between rigid as shown in **a** and elastic walls in **b** for the carotid artery

##### At the blood vessel wall

A no-slip boundary condition is adopted for the guidewire and the blood vessel walls. To fully model the blood flow, a further research is performed in our lab to compare the predicted results of rigid and elastic arterial wall of the carotid artery. The arterial wall elasticity through two-way fluid–structure interaction (FSI) was considered to calculate the blood flow dynamic factors. Predicted results indicated that there is no significant difference in the predicted dynamic factors between elastic and rigid walls as shown in. Additionally, for one case, the computational time for considering the FSI procedure was about 15 days compared to 6 h under rigid wall assumption. Accordingly, in the current study, the arterial wall is assumed to be rigid (Fig. 11).

##### At the inlet

**Fig. 12:**
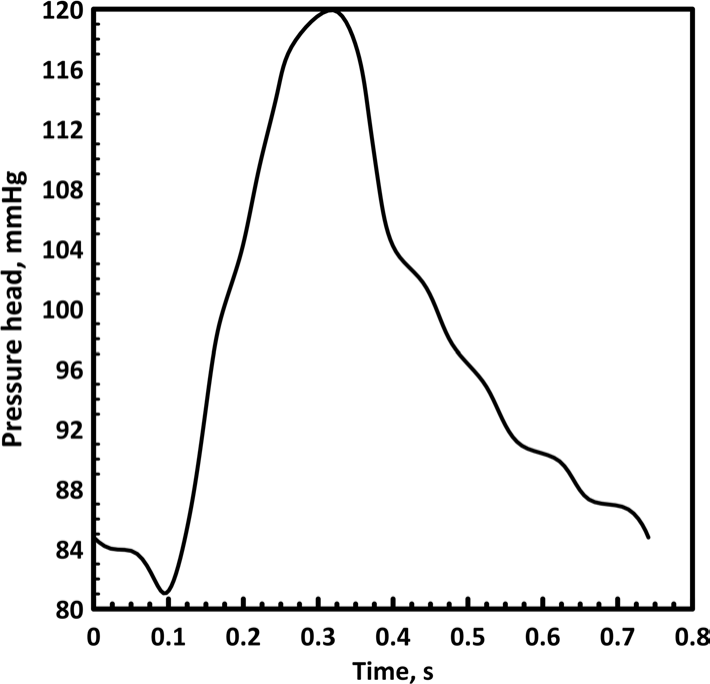
Inlet pressure distribution

The aortic pressure profile shown in Fig. 12 is considered at the inlet (from Saraiva et al. [43]). This pressure profile is implemented using the transient table.

##### At outlets

At the end of the arterial tree, the pressure is constant with a value of ${{\varvec{P}}}_{{\varvec{v}}}=0$ gauge [10]. In the present simulations, part of the arterial tree could not be obtained from the CT scans. Accordingly, it is assumed that the geometry ends at this point neglecting the remaining part of the arterial tree in all simulations. Accordingly. at the outlets, the pressure is assumed to be zero gauge [43].

##### Initial conditions

The initial values of pressure and all velocity components are set to zero.

### Numerical simulation

To investigate the effect of placing guidewire in stenotic coronary artery, the governing equations along with boundary and initial conditions are numerically simulated using computational fluid dynamics (CFD) commercial package (ANSYS FLUENT 19.1 code). The governing partial differential equations are discretized using the finite volume method. The discretized equations are solved using a fully implicit scheme with second order upwind spatial differences. The SIMPLE algorithm for the coupling of pressure and velocities is used to accomplish this. A Dell Precision T7500 workstation with an Intel Xeon® processor 3.75 GHz, 48 cores, and 64 GB installed memory is used to implement parallel computing. In addition, the grid and time step independence tests are carried out as described in the subsection below.

#### Grid and time step-independent tests

**Fig. 13:**
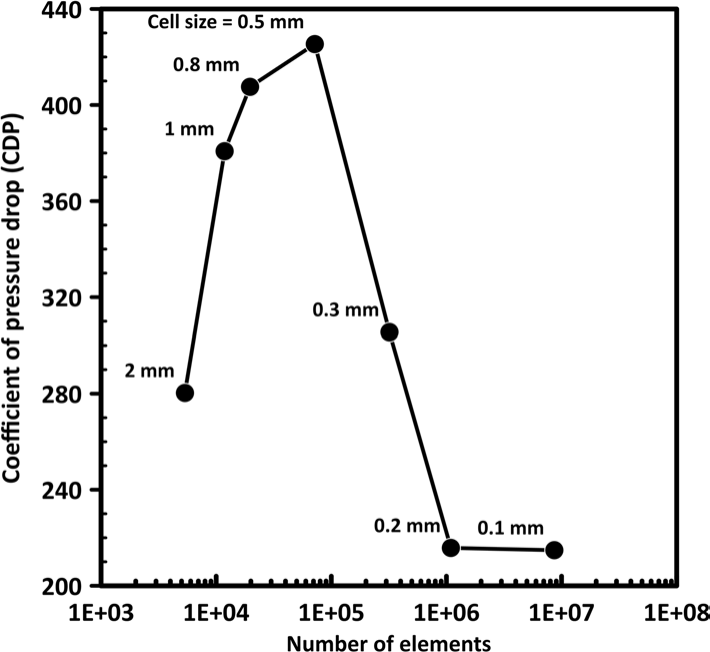
Mesh-independent test

Grid independent test is conducted on the stenosis model and the maximum utilized cell sizes are 2, 1, 0.8, 0.5, 0.3, 0.2 and 0.1 mm which are corresponding to 5,347, 11,844, 19,650, 71,893, 319,734, 1,093,264 and 8,666,800 cells, respectively. It is found that at the size of the element 0.2 mm (1,093,264 cells), the CDP value is observed to be independent of the number of grids, as shown in Fig. 13. Accordingly, the optimum cell size is 0.2 mm for all the numerical simulations.

**Fig. 14:**
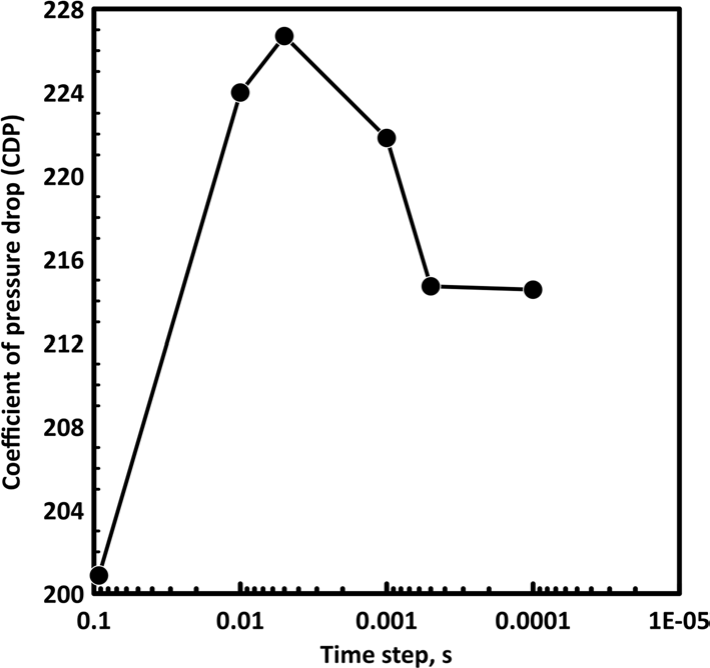
Time step-independent test

Different time steps are tried in this simulation. When the time step reaches 0.0005 s, the effect of decreasing the time step on the CDP is reduced. Accordingly, a time step of 0.0005 s is used for all the simulated models (Fig. 14).

### Model validation

In the current work, an experimental setup was established to measure the flow rate through the RCA. The main setup components are a reservoir, shut-off valve, hose and two collecting tanks. Experiments are performed using a reservoir with water level of 1.20 m above the inlet. The reservoir diameter is large (0.5 m) to keep the inlet pressure constant. Water is used as a working fluid for its availability and safety. The 3-D geometries obtained from CT scans are printed using a 3D printer. The shut-off valve is attached to the reservoir to control starting and stopping the experiments. The hose is connected between the shut-off valve and the inlet of the geometry. The outlet flow rate from each branch is measured using a collecting tank.

The experiment procedure is as follows:

- Connect the first 3D printed model for the RCA with the hose from the reservoir.
- Add water to the reservoir to keep the level at 1.2 m from the outlets.
- Open the shut-off valve to begin the experiment for 30 s and collect the water from each outlet in the collecting tanks.
- Repeat the experiment 10 times and take the average flowrate from each outlet.
- Replace the geometry and repeat the experiment.

**Fig. 15:**
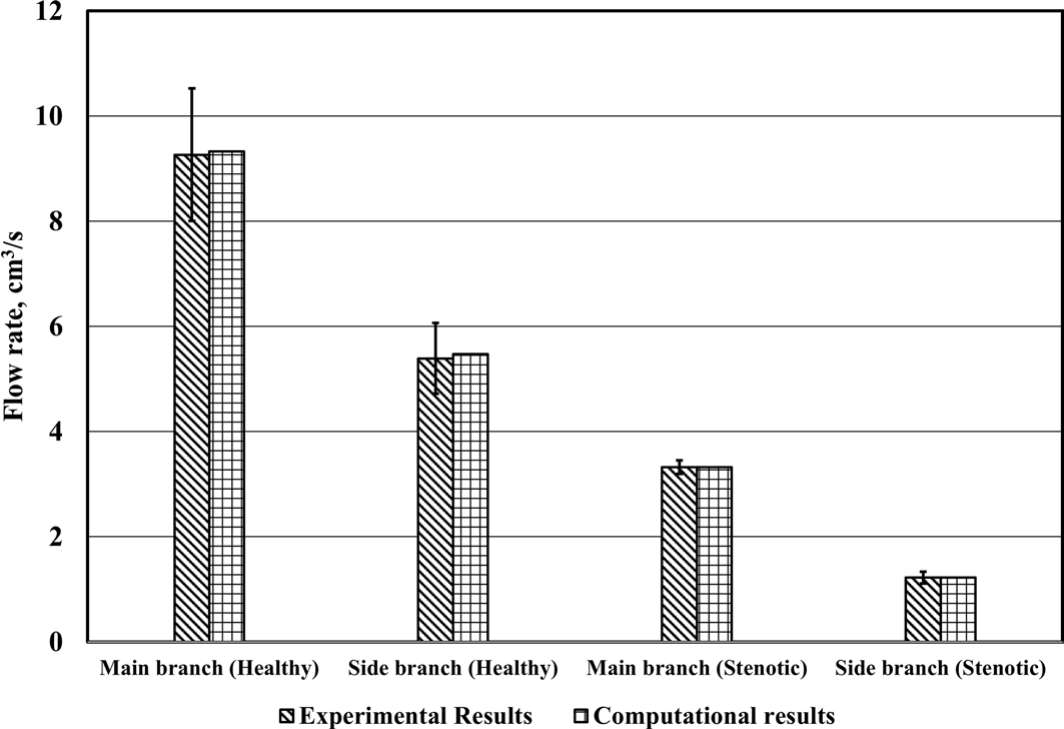
Comparison between experimental and numerical results for healthy artery

In case of a healthy artery, the volume flow rate that directed towards the main branch is 9.26 cm3/s representing 63.2% of the total flow delivered from the main branch. The remaining 36.8% is diverted to the secondary branch. To validate the model, Fig. 15 shows the comparison between experimental and computational flow rates in the main and side branches for all cases. Based on figures, a good agreement between the measured and predicted values of flow rates are shown. The maximum deviation between the measured and predicted values is about 1.5%. This is mainly due to the experimental measurement error.

## Supplementary Information

**Additional file 1: Appendix S1**. Analysis of non-Newtonian models.

## Data Availability

Not available.

## Abbreviations

CAD: Coronary artery disease
IVUS: Intravascular ultrasound
FFR: Fractional flow reserve
CT: Computed tomography angiography
CFD: Computational fluid dynamics
FFRCT: Computed tomography fractional flow reserve
RCA: Right coronary artery
CDP: Pressure drop coefficient
DICOM: Digital Imaging and Communication in Medicine
SCCT: Society of Cardiovascular Computed Tomography
