# Supplementary material for: Effect of guidewire insertion in fractional flow reserve procedure for real geometry using computational fluid dynamics
Source: Biomed Eng Online. 2021 Sep 28;20:95. doi: 10.1186/s12938-021-00935-y (PMC8479905; doi:10.1186/s12938-021-00935-y)
Supplement: Supplementary file 1 — Additional file 1: Appendix S1. Analysis of non-Newtonian models. [file 12938_2021_935_MOESM1_ESM.docx]

# Appendix A

Blood is a shear-thinning fluid which behaves differently with the shear rate. To select the most accurate non-Newtonian model, Newtonian and four non-Newtonian models were firstly applied to validate the numerical simulation, hence determine the appropriate viscosity model. The most common non-Newtonian models are the Power-law, Carreau, Cross and Quemada models [1,2].

The predicted results are compared with clinical data. However, it is difficult to measure velocity inside coronary arteries clinically. Consequently, the clinical data is not available in case of coronary artery. Hence, the carotid artery geometry is used in selecting the most appropriate non-Newtonian model, the geometry is obtained using 3D reconstruction of CT Scan as shown in Fig. X and the velocity profile inside the common carotid artery and inside the internal carotid artery is monitored using ultrasonic doppler. The blood flow is simulated using five blood models (Newtonian, Power-Law, Carreau, Quemada and Cross).

The Carreau model is one of the common and simplest non-Newtonian models which describe the blood behavior. the Carreau model is representing the relation between the dynamic viscosity ($\mu)$ and strain rate ($\dot{\gamma}$) as following [3]:

$\mu=\mu_{\infty}+\left( \mu_{o}-\mu_{\infty} \right)\times\left[ 1+\left( \lambda\dot{\gamma} \right)^{2} \right]^{\frac{n-1}{2}}$ (4)

Where, $\mu_{0}$and $\mu_{\infty}$ are zero shear rate viscosity and infinite shear rate viscosity $(Pa\cdot s)$ respectively, $n$ is the power index, $\lambda$ is the time constant $(s)$ and $\dot{\gamma}$ is the shear rate ($s^{-1}$).

The power-law non-Newtonian model is similar to the Carreau model and described as the following [4]:

$\mu_{min}> \mu=k\dot{\gamma}^{n-1}< \mu_{max}$ (5)

where, $\mu_{min} and \mu_{max}$ are the minimum and maximum viscosities ($pa\cdot s$), $k$ is the flow consistency index, $n$ is the power-law index and $\dot{\gamma}$ is the shear rate ($s^{-1}$).

The cross non-Newtonian model is defined as the following [5]:

$\mu=\mu_{\infty}+\frac{\left( \mu_{o}-\mu_{\infty} \right)}{1+\left( \frac{\dot{\gamma}}{\gamma_{c}} \right)^{n}}$ (6)

where, $\mu_{0}$and $\mu_{\infty}$ are zero shear rate viscosity and infinite shear rate viscosity $(Pa\cdot s)$ respectively, $n$ is the model constant, $\gamma_{c}$ is the reference shear rate ($s^{-1}$), and $\dot{\gamma}$ is the shear rate ($s^{-1}$).

Meanwhile, the Quemada model in described as the following [6]:

$\mu=\mu_{f} \left[ 1-0.5 \frac{k_{0}+k_{\infty}\sqrt{\frac{\left\| \dot{\gamma} \right\|}{\gamma_{c}}}}{1+\sqrt{\frac{\left\| \dot{\gamma} \right\|}{\gamma_{c}}}} \phi\right]^{-2}$ (7)

Where, $\mu_{f}$is the plasma shear rate viscosity $(Pa.s)$, $k_{0}$ and $k_{\infty}$ are the maximum volume fraction for zero and infinite shear rate, $\phi$ is the blood volume concentration, $\gamma_{c}$ is the characteristic shear rate ($s^{-1}$), and $\dot{\gamma}$ is the shear rate ($s^{-1}$).

The Newtonian and these constitutive models’ parameters are summarized in table 1.

**TABLE 1:** BLOOD RHEOLOGY MODELS PARAMETERS

| Model | Parameter |
| --- | --- |
| Carreau | $\mu_{\infty}=0.00345 Pa.s$ |
|  | $\mu_{0}=0.056$ Pa. s $\lambda=3.131 s$ $n=0.3568$ |
| Power-law | $k=0.01467$ $n=0.7755$ $\mu_{min}=0.00345 Pa.s$ $\mu_{max}=0.025 Pa.s$ |
| Cross | $\mu_{0}=0.00364 Pa.s$ $\gamma_{c}=2.63 s^{-1}$ $n=1.45$ |
| Quemada | $\mu_{f}=0.0012 Pa.s$ $\gamma_{c}=1.88 s^{-1}$ $k_{0}=2.07$ $k_{\infty}=4.33$ $\phi=0.45$ |

The predicted value of velocity inside the internal carotid artery is compared with the clinical data. Fig. 1 shows the comparison between the obtained numerical and measured velocity.


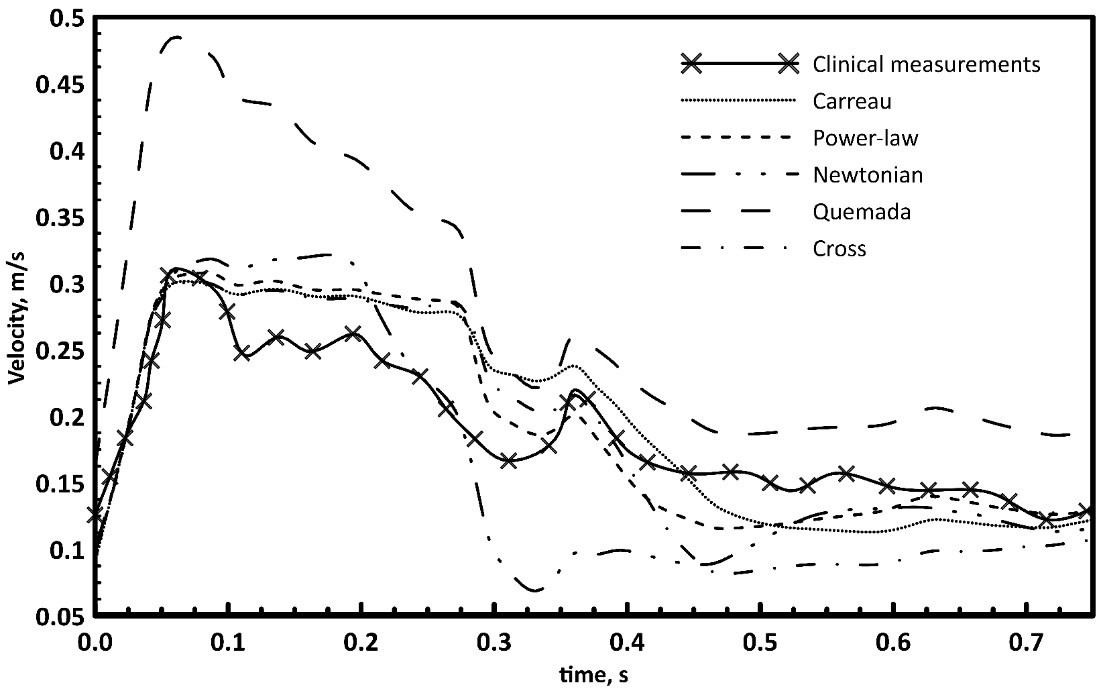


Figure 1 Comparison between five blood models and the clinical measurements for the velocity inside the internal carotid artery

Fig. 1 shows that power-law and Carreau model are the closest to the measured data. Table 2 represents the time-averaged error between the predicted and the measured values. It shows that the best model is Carreau model and the worst model is Quemada. Accordingly, the Carreau model is used in all simulations in the present work.

Table 2 Errors for different blood models

|  | Average error (%) | Maximum error (%) |
| --- | --- | --- |
| Newtonian | 3.17 | 19.87 |
| Power-Law | 0.83 | 18.51 |
| Carreau | 0.5 | 17.87 |
| Quemada | 7.5 | 36.28 |
| Cross | 2.1 | 17.92 |

**REFERENCES**

[1] A. Skiadopoulos, P. Neofytou, C. Housiadas, Comparison of blood rheological models in patient specific cardiovascular system simulations, J. Hydrodyn. 29 (2017) 293–304.

[2] J.B. Mendieta, D. Fontanarosa, J. Wang, P.K. Paritala, T. McGahan, T. Lloyd, Z. Li, The importance of blood rheology in patient-specific computational fluid dynamics simulation of stenotic carotid arteries, Biomech. Model. Mechanobiol. (2020) 1–14.

[3] Y.I. Cho, K.R. Kensey, Effects of the non-Newtonian viscosity of blood on flows in a diseased arterial vessel. Part 1: Steady flows, Biorheology. 28 (1991) 241–262.

[4] M.A. Hussain, S. Kar, R.R. Puniyani, Relationship between power law coefficients and major blood constituents affecting the whole blood viscosity, J. Biosci. 24 (1999) 329–337.

[5] M.M. Cross, Rheology of non-Newtonian fluids: a new flow equation for pseudoplastic systems, J. Colloid Sci. 20 (1965) 417–437.

[6] D. Quemada, Rheology of concentrated disperse systems III. General features of the proposed non-newtonian model. Comparison with experimental data, Rheol. Acta. 17 (1978) 643–653.
